# The Effect of *Lactobacillus plantarum* on the Fecal Microbiota, Short Chain Fatty Acids, Odorous Substances, and Blood Biochemical Indices of Cats

**DOI:** 10.3390/microorganisms12010091

**Published:** 2024-01-02

**Authors:** Bing Han, Shukun Liang, Jintao Sun, Hui Tao, Zhenlong Wang, Baosheng Liu, Xiumin Wang, Jie Liu, Jinquan Wang

**Affiliations:** 1 Key Laboratory of Feed Biotechnology of Ministry of Agriculture and Rural Affairs, Institute of Feed Research, Chinese Academy of Agricultural Sciences, No. 12 Zhong Guan Cun South Street, Haidian District, Beijing 100081, China; kongkong@cau.edu.cn (S.L.); taohui@caas.cn (H.T.); wangzhenlong02@caas.cn (Z.W.);; 2 School of Veterinary Medicine, China Agricultural University, Beijing 100193, China; 3 College of Animal Science and Technology, Jiangxi Agricultural University, Nanchang 330045, China

**Keywords:** *Lactobacillus plantarum*, fecal microbia, cats, SCFA, sIgA, indole

## Abstract

Lactobacilli have played an important role in the gut health of pets. The aim of this research was to study the effects of isolated Lactobacilli (named L11) on the immune, nutrient metabolism, and gut health of cats. Twelve healthy adult cats were randomly assigned into two groups, the control group (CONTROL, *n* = 6, without any probiotics product) and the treatment group (probiotics, *n* = 6, L11 10^9^ CFU/kg feed), while using the same dry diet. On day 28, blood and fecal samples were collected, and the blood biochemical indices, fecal microbiota, short-chain fatty acids (SCFAs), immunological parameters, and odorous substances were separately tested. The triglyceride of the blood was decreased after using L11 (*p* < 0.05), which could probably alleviate the occurrence of cat obesity to some extent. The sIgA of the feces was increased by 30.1% (*p* < 0.05), which could enhance the cat’s immunity. The abundance of *Bifidobacteria* was increased after using L11 (*p* < 0.05), and the indole and 3-methylindole of the feces were both reduced compared with the control group; 3-methylindole was especially reduced by 67.3% (*p* < 0.05), which showed that L11 could also improve the intestinal state of cats. Therefore, this research shows that L11 could be a good choice to improve the gut health and immune functions of cats, and it is probably related to the lipid mechanism of cats.

## 1. Introduction

Probiotics are a kind of live and beneficial microorganism [1]; they are now widely used on companion animals clinically, mainly dogs and cats, and it has been shown that probiotics have positive effects on pets in IBD treatment [2], acute gastroenteritis [3], prevention of allergies [4], and so on.

The constitution of the gut microbiota of cats mainly includes four phylum, including Firmicutes, Actinobacteria, Bacteroidetes, and Actactabacteria [5]. Among the lactic acid bacteria, it has been shown that Lactobacilli play an important role in the gut of dogs [6,7,8,9], but the research of Lactobacilli on cats has been very limited; only one research study about *Lactobacillus acidophilus* was performed on cats [10].

Lactobacilli (L.), belonging to one of the probiotics, plays a dominant part in the gut of pets, including canines and felines. Now, more related studies, mainly on canines, showed that Lactobacilli widely exist in the canine GIT and could adjust the gut microecosystem [11]. Three Lactobacilli bacteria (including *L. rhamnosus* VET 16 A, *L. fermentum* VET9A, and *L. plantarum* VET14A) from canines showed that Lactobacilli could adhere to the gut mucus and possibly prevent the adhesion of pathogens, like *Enterococcus. coli* (*E. coli*), *Salmonella enteria* (*S. enteria*), and so on [12]. Also, *L. acidophilus* DSM13241 was proved to be steady in the canine GIT, and could increase the Lactobacilli and decrease Clostridia in feces after using it, and even improve the immune parameters [6]. The above studies showed that Lactobacilli could be very important and benefit to canines, but there has been very little studies on cats compared with canines.

The aim of this research was to study the effects of the isolated probiotic on the immune, nutrient metabolism, and gut health of cats and that this study would supply more theories for the application of probiotics on cats.

## 2. Materials and Methods

### 2.1. The Screening and Cultivation of the Bacteria

Cat feces samples were collected and enriched in MRS broth (1% peptone, 0.5% beef powder, 2% glucose, 0.4% yeast barm) (Solarbio, Beijing, China) for 48 h without vibration. The fermentation was then inoculated onto MRS soft agar plates (1% agar, *w*/*v*), and a single white colony (named L11) was screened. The 16S rRNA of this isolated sample was identified. L11 was inoculated in MRS liquid culture medium at 37 °C for 48 h for secondary purification, and the secondary purification ensured its purity and stability. After that, the secondary purified product was frozen at −80 °C for subsequent experiments, and the L11 bacterial liquid was centrifuged (12,000× *g*/min, 4 °C, 10 min). 

### 2.2. Inhibition of the Bacteria against Pathogens

The agar diffusion method was first used to test the inhibition on pathogens of the isolate [13]. First, the inhibitory effect of L11 on pathogens was tested by the agar diffusion method [13]. *E. coli* CVCC 1555, *E. coli* ATCC 14028, *S. aureus* ATCC 43300, and *S. enteritidis* CVCC 3377 were selected as indicators. All pathogens were grown in NA medium (nutrient agar, including 1% peptone, 0.3% beef powder, 0.5% NaCl) (Solarbio, Beijing, China) and incubated at 37 °C for 24 h. The culture of pathogenic bacteria was inoculated (200 µL) into fresh NA soft agar plates (1% agar, *w*/*v*). All plates were incubated at 37 °C for 24 h, and the diameter of the antibacterial circle around the wells was measured. 

### 2.3. Experimental Animals and Sample Collection

L11 was cultured and was preserved at the China General Microbiological Culture Center, the deposit number of which was *Lactobacillus plantarum* CGMCC 24558. Then, the fermentation of L11 (MRS liquid medium, 37 °C, 48 h) was first centrifuged (6000× *g*/min, 10 min) and concentrated with lyophilizer (Sihuan Bioengineering Co., Ltd., Beijing, China) to the bacterial powder (10^12^ CFU/g) for the animal test.

Twelve healthy adult cats (all about 2 years old, female) were randomly assigned into two groups, the control group (CONTROL, *n* = 6, without any probiotics product) and the treated group (probiotics, *n* = 6, 10^9^ CFU/kg feed), while using the same dry diet. The test was implemented for 28 days. The probiotics were administrated with food every day. The cats were housed in twelve stainless steel cages (162 × 68 × 189 cm), and the temperature of the room was about 25 ± 2 °C. Each was furnished with litter boxes and food bowls. Fresh water was available all the time. Cats were fed once at the same time every day. On day 28, the feces were collected from each section of fresh feces for the microbial abundance analysis and stored at −80 °C. At the same time, blood samples were collected for biochemical testing. All treated felines did not use any antibiotics and probiotics during testing.

### 2.4. Blood Biochemical Test

In this experiment, the blood was centrifuged after being collected. A biochemical analyzer (MNCHIP, Tianjin, China) was used to test four biochemical parameters of the serum of the blood, including total bilirubin (TBIL), total bile acid (TBA), triglyceride (TG), and cholesterol (CHOL) [14]. 

### 2.5. The Test of sIgA (Secretory Immunoglobulin A) of the Feces

SIgA is a kind of defensive protein in the body of animals. In this research, the sIgA of the feces of day 28 was tested with the ELISA method (Jiangsu Meimian Industrial Co., Ltd., Yancheng, China). Cat sIgA levels in the samples were tested using purified cat sIgA antibodies to coat microtiter plate wells, making solid-phase antibodies, and then adding sIgA to the wells. The reaction was terminated by the addition of a sulfuric acid solution, and the color change was measured spectrophotometrically at a wavelength of 450 nm. The concentration of sIgA in the samples was then determined by comparing the O.D. of the samples with the standard curve. 

### 2.6. The Test of Short-Chain Fatty Acids of the Feces and Fermentation Supernatant

The short-chain fatty acids (SCFAs) of the feces samples of day 28 were tested using ion chromatography (Metrohm, Herisau, Switzerland). Similarly, L11 was inoculated in MRS liquid culture medium at 37 °C, and the supernatant was separated at 24 h and 48 h. The culture was centrifuged (12,000× *g* for 10 min at 4 °C), and the cell-free supernatant was sterilized by filtration through a 0.22 µm pore size filter (Millipore, MA, USA). [14].

### 2.7. The Test of the Indole and 3-Methylindole of the Feces

The fresh feces on day 28 (2 g of each sample) was first extracted in 10 mL of methyl alcohol and then placed in a water bath at 40 °C for 20 min. Then, the supernatant of each sample was centrifuged at 11,612× *g* for 10 min and used to test the indole and 3-methylindole present using the HPLC (SHIMADZU, Kyoto, Japan) method. The indole and 3-methylindole were both detected at 263 nm.

### 2.8. The Indentification of Bacteria Using 16S rDNA

The 16S rDNA was extracted by PCR and high-throughput sequencing at Sangon Biotech Co., Ltd. (Shanghai, China), and blasted on the NCBI (National Center of Biotechnology Information) database.

### 2.9. Extraction of Fecal DNA 

Total DNA was extracted from fecal samples from day 28. An E.Z.N.A Mag-Bind Soil DNA Kit (Omega, M5635-02, San Antonio, TX, USA) and Quibit dsDNA HS kit (Thermo, Waltham, MA, USA) were used to test the concentration of DNA samples. The extracted DNA samples were stored at −70 °C and used for further PCR amplification [9].

### 2.10. PCR Amplication for Amplicon 

The PCR products were checked using electrophoresis. The V3–V4 of the 16S rDNA were amplified and sequenced by second-generation sequencing technology at Sangon Biotech Co., Ltd. (Shanghai, China).

The sequence of the forward primer was CCTACGGGNGGCWGCAG, and the reverse primer was GACTACHVGGGTATCTAATCC. PCR testing was amplified twice. The first PCR reaction conditions referred to the method [9]. 

### 2.11. Data Analysis

The alpha diversity indices were identified in terms of the OTU richness of fecal microbiota. Alpha diversity indices were calculated using Mothur (version 1.43.0). Beta diversity was used to evaluate the differences in the microbiome among samples and was normally combined with dimensional reduction methods such as principal coordinate analysis (PCoA) to obtain visual representations. A difference comparison was used to identify features with significantly different abundances between groups using STAMP (version 2.1.3) and LefSe (version 1.1.0) software. The correlation coefficients and *p*-values between communities/OTUs were calculated using SparCC (version 1.1.0). An evolution tree was constructed using Mega (version 7.0.26).

The data of blood, SCFAs, and some fecal indices (sIgA, indole etc.) were analyzed with a one-way ANOVA test followed by Tukey’s multiple range test; data were expressed as the mean ± SE by Tukey’s multiple range test, and it was expressed as significant if *p* was less than 0.05. These related statistical analyses were performed using SPSS 25.0 (SPSS Inc., Chicago, IL, USA) software.

## 3. Results

### 3.1. The Screening and Identification of the Bacteria

**Figure 1:**
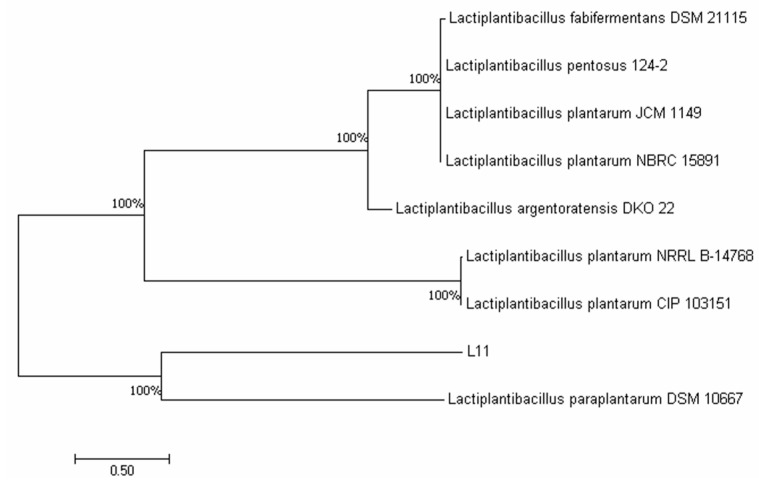
Construction of the evolutionary tree for L11.

The bacteria, named L11, was isolated from cat feces and cultured in MRS broth at 37 °C for 24h, and the 16S rDNA was extracted using Mega 7, as shown in Figure 1.

### 3.2. Inhibition Characterization of L11

The inhibition for pathogens was implemented using the agar diffusion method, and the results are shown in Table 1. From Table 1, we could conclude that L11 showed good inhibition abilities for pathogens. So, we chose these bacteria for the animal test to check the function and safety of the bacteria.

### 3.3. Blood Biochemical Indexs

**Figure 2:**
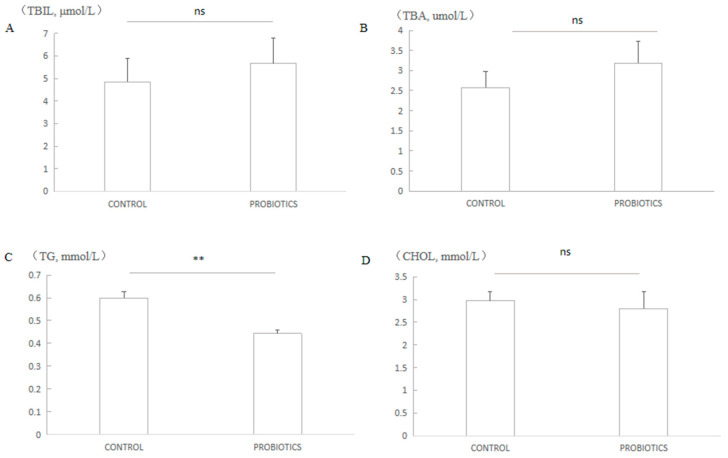
The effect of probiotics on the blood biochemical indices. ns means *p* > 0.05; ** means *p* < 0.01. (**A**) The serum concentration of TBIL of different treatments; (**B**) The serum concentration of TBA of different treatments; (**C**) The concentration of TG in serum; (**D**) The concentration of CHOL in serum.

The blood biochemical indices, including total bilirubin (TBIL), total bile acid (TBA), triglyceride (TG), and cholesterol (CHOL), were tested using the biochemical analyzer (MNCHIP Technologies Co., Ltd., Tianjin, China). The triglyceride on probiotics treatment was decreased by 20.9% compared with the control treatment (*p* < 0.01) (Figure 2C). There was no significance for the other three biochemical indices compared with the control treatment (*p* > 0.05) (Figure 2A,B,D).

### 3.4. The Test of sIgA of Feces

**Figure 3:**
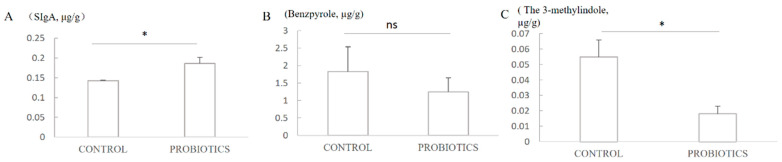
(**A**) The effect of probiotics on the sIgA of the feces; (**B**) the effect of probiotics on the concentration of indole of the feces; (**C**) the effect of probiotics on the concentration of the 3-methylindole of the feces. ns means *p* > 0.05; * means *p* < 0.05.

The sIgA of feces was tested using the ELISA method, and the result showed that the sIgA of the probiotics group was improved by 30.1% (*p* < 0.05) compared with the control group (Figure 3A).

### 3.5. The Indole and 3-Methylindole of the Feces

The indole and 3-methylindole of the feces were tested using the HPLC method. As shown in Figure 3B,C, the indole of the L11 treatment was reduced by 31.8% compared with the control treatment (*p* > 0.05), and the 3-methylindole was reduced by 67.3% after using L11 (*p* < 0.05).

### 3.6. SCFAs Concentration 

Six kind of SCFAs from the feces samples of day 28 were tested using ion chromatography (Metrohm, Swiss, Herisau, Switzerland). In Table 2, only propionic acid was decreased with significance (*p* < 0.05), and there was no significance between two groups of other SCFAs (*p* > 0.05).

### 3.7. Fecal Microbiota Analysis 

**Figure 4:**
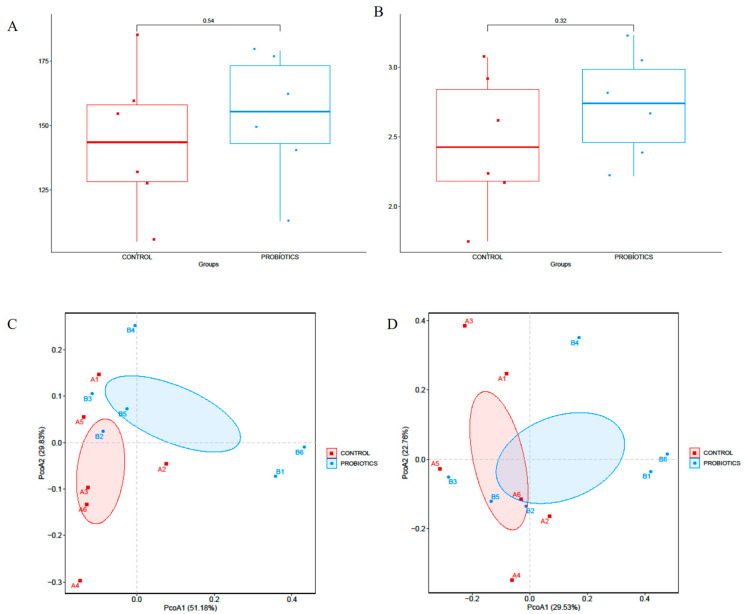
(**A**) The OTUs of the microbial community; (**B**) Shannon’s indices of the microbial community; (**C**) principal coordinate analysis (PCoA) of the phylum level; A1–A6 meant the number of the animals of control groups, and B1–B6 meant the number of the animals of probiotics groups; (**D**) principal coordinate analysis (PCoA) of the genus level.

The fecal samples of the felines were sequenced. The alpha diversity of the microbial community was measured using the OTUs and Shannon’s indices; but there was no significant differences between the two groups in the diversity of the microbial community (*p* > 0.05) (Figure 4A,B). Beta diversity evaluates the differences in the microbiome among samples and is normally combined with dimensional reduction methods such as principal coordinate analysis (PCoA) to obtain visual representations (Figure 4C,D); Figure 4C shows the phylum level, which showed the apparent differences between the control group and probiotics group (*p* < 0.05), while Figure 4D showed that there was no significance between the control group and probiotics group (*p* > 0.05). 

**Figure 5:**
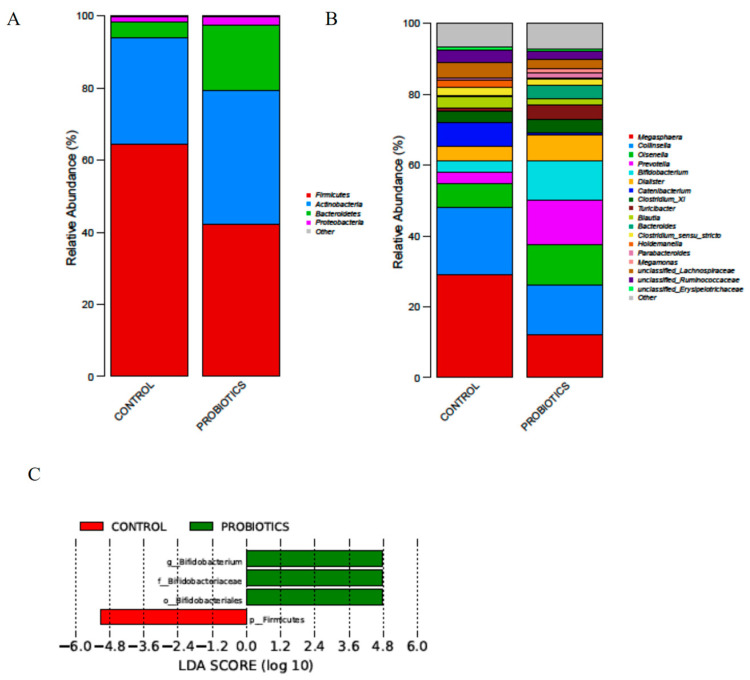
Analysis of diversified species of fecal microbiota. (**A**) The figure shows the abundance of the phylum level of changes in the fecal microbiota. (**B**) The figure shows the genus level of the changes in the fecal microbiota. (**C**) The figure shows the genus LDA score of the two treatments.

After supplementation of L11, on the phylum level, the abundance of the *Firmicutes* was decreased and the abundance of *Bacteroidetes* and *Actinobacteria* was increased (*p* < 0.05) (Figure 5A). On the genus level, the abundance of *Bifidobacterium*, *Lactobacillus*, and *Megasphaera*, the core Lactobacillus in the gut, were all increased after using L11 (Figure 5B), of which the abundance of *Bifidobacterium* was increased (*p* < 0.05) (Figure 5C).

## 4. Discussion

The research showed the partial characterization and function of *Lactobacillus plantarum* L11 isolated from the feces of cats in vivo and in vitro and showed the positive effect on the modulation of fat and gut, which were also found to be positive effects in these studies [15,16,17].

In vitro, the bacteria L11 showed good inhibition activity against several pathogens, and the SCFAs and polysaccharides could probably be the reasons for the inhibition ability against pathogens, which was the same as most lactic acid bacteria [18].

In vivo, the function of the L11 on the blood parameters, fecal microbiota, immunological parameter, and skatole of the cats were separately studied. The L11 could reduce the blood triglyceride by 20.6%, and this result was the same as former studies [19], which showed that the bacteria could modulate the fat metabolism of cats to some extent. Now, many studies have shown that *Lactobacillus plantarum* could modulate the fat metabolism, especially in high-fat diets. The mechanism of how to modulate has been studied [20,21], and the mechanism of this bacteria in this research was not studied yet, which will be focused on in future.

Immunity was also very important for animals, and it has been proved that Lactobacilli could modulate the immunity ability of different animals [22,23,24]. In this research, sIgA in the feces was studied to find out whether L11 could modulate the immunity ability of cats. After using L11, the concentration of sIgA was improved by 30.1%, which was in accordance with the former studies [25]. 

The composition of the microbiota of cats was related with the age, gender, feed, body health, type, and even area of cats. Some studies have shown that Firmicutes possessed the majority position, and Actinobacteria and Bacteroides were much less than Firmicutes [26], which was in agreement with our results of the control treatment. But after using L11, the composition of microbiota was greatly changed on the phylum level (*p* < 0.05); the abundance of Firmicutes was greatly reduced, and the abundance of Actinobacteria and Bacteroides were increased (*p* < 0.05). Meanwhile, the Firmicutes/Bateroides were greatly decreased, which has been showed to be related with complex carbohydrates and protein ratios, previously observed in dogs [27]. The *Bifidobacterium*, an important benefit group of the Actinobacteria phylum, was greatly increased so that the gut health of cats was improved. This result was also shown in other studies [28]. The abundance of *Prevotella* and *Bacteroides* were both increased, which was in accordance with the former studies [29]. *Prevotella* was related with the carbohydrates metabolism, and Bacteroides was related with protein and fat metabolism [30]. For the high protein and fat in the diet, *Bacteroides* was very important for cats. This could prove that L11 could modulate the fat metabolism by increasing the abundance of the related genus in the gut. 

The content of SCFAs was related to the main genus of the gut [31]. In this research, SCFAs were not affected by the bacteria except for propionic acid. The decrease in propionic acid was probably due to the change of the gut microbiota composition, but the closely related bacteria require further study.

The indole and 3-methylindole in the feces were closely related to protein content in the diet [32], which have caused more attention for humans. This is because cats are meat-eating animals, and the protein content in the feed was often higher than the standard; therefore, the digestibility of protein usually could not be 100%, and there would be some odorous matter, like indole and 3-methylindole, existing in the feces. The research showed that after using L11, the 3-methylindole was reduced by 67.3% after using L11 (*p* < 0.05), which was also proven in dogs [33]. The reason was probably because the protein digestibility was improved for the changing of gut microbiota. 

## 5. Conclusions

The research showed that blood triglyceride was decreased after using L11 (*p* < 0.05). The abundance of *Bifidobacteria* was increased after using L11 (*p* < 0.05). The sIgA of the feces was increased by 30.1% (*p* < 0.05). The 3-methylindole was reduced by 67.3% (*p* < 0.05). These results all show that the bacteria L11 could be related to the fat metabolism of cats and affect the gut microbiota positively so that the odorous substances decrease for the improvement of the digestibility of nutrients. This paper supplied theories for the application of L11 on cats.

## Figures and Tables

**Table 1:** The inhibition for pathogens of L11.

Strain	Inhibition Effect
*E. coli* CVCC 1555	++
*E. coli* ATCC 14028	++
*S. aureus* ATCC 43300	++
*S. enteritidis* CVCC 3377	+

Notes: The inhibition zone around the Oxford cup containing the supernatant of the bacteria fermentation broth was classified as ++, 12–15 mm; +, 9–11 mm.

**Table 2:** The SCFAs of the feces.

SCFAs	CONTROL	PROBIOTICS	*p* Value
Acetic acid	1.57 ± 0.11	1.27 ± 0.27	0.202
Propionic acid	0.59 ± 0.02 ^a^	0.39 ± 0.06 ^b^	0.011
Butyric acid	0.25 ± 0.09	0.28 ± 0.01	0.757
Valeric acid	0.19 ± 0.06	0.16 ± 0.02	0.356
Isobutyric acid	0.05 ± 0.01	0.07 ± 0.03	0.242
Isovaleric acid	0.07 ± 0.02	0.11 ± 0.05	0.288

Note: Different superscripts in the table denote significant differences (*p* < 0.05).

## Data Availability

Data are contained within the article.
